# Burden of migraine among Japanese patients: a cross-sectional National Health and Wellness Survey

**DOI:** 10.1186/s10194-020-01180-9

**Published:** 2020-09-10

**Authors:** Shoji Kikui, Yirong Chen, Hiroshi Todaka, Keiko Asao, Kenji Adachi, Takao Takeshima

**Affiliations:** 1 Department of Neurology & Headache Center, Tominaga Hospital, 1-4-48 Minatomiachi, Naniwa-ku, Osaka, 556-0017 Japan; 2 Kantar, Health Division, 50 Scotts Road, #02-01, Singapore, 228242 Singapore; 3 Amgen K.K., Midtown Tower, 9-7-1 Akasaka, Minato-ku, Tokyo, 107-6239 Japan

**Keywords:** Migraine, Health-related quality of life, Work productivity, Healthcare resource utilization, Costs, Economic burden

## Abstract

**Background:**

Limited studies have measured the burden of migraine in Japan. This study aimed at estimating the disease burden of migraine in Japan and identifying factors associated with the burden using the 2017 National Health and Wellness Survey.

**Methods:**

Migraine patients were defined by ICHD-3 like criteria with ≥4 monthly headache days (MHDs), and non-migraine respondents were selected using 1:4 propensity score matching. Multivariate analyses were conducted to compare Health-related Quality of Life (HRQoL), work productivity and activity impairment (WPAI), healthcare resource utilization (HRU) and costs between the two groups, and to identify factors associated with these outcomes in migraine patients.

**Results:**

In 30,001 respondents, 378 migraine patients were identified. Compared to matched controls (*N* = 1512), migraine patients had lower physical (45.17 vs. 49.89), mental (42.28 vs. 47.71) and role/social (37.91 vs. 44.19) component summary scores (*p* < 0.001). Migraine patients had higher absenteeism (6.4% vs. 2.2%), presenteeism (40.2% vs. 22.5%), total work productivity impairment (44.3% vs. 24.5%), total activity impairment (45.0% vs. 23.9%), indirect costs (1,492,520 JPY vs. 808,320 JPY) and more visits to healthcare providers in the past 6 months (7.23 vs. 3.96) (*p* < 0.001). More MHDs was associated with worse HRQoL, and higher HRU and indirect costs.

**Conclusions:**

Japanese migraine patients experience an incremental burden. This demonstrates the unmet needs among Japanese migraine patients.

## Background

The importance of understanding the burden of illness associated with migraine is becoming increasingly recognized, both as part of clinical trials and health technology assessment [1]. This includes measuring the burden of disease by use of patient reported outcome tools such as health-related quality of life (HRQoL), the impact of illness on work productivity and the associated economic burden [1]. The evaluation of how migraine affects patients’ lives from their perspective is useful for identifying therapeutic areas of need and providing guidance for development of future treatment [2, 3].

It has been well established that migraine is associated with burden for both patients and society worldwide [4–7]. The global burden of disease calculation from 2016 ranked migraine as the second leading cause of years of life lived with disability (YLD) accounting for 5.6% of total YLD, following lower back pain [4]. A narrative review identified six main themes around burden and impact of migraine, including the prevalence, overall impact, impact on work or school activities, impact on family, interictal burden, and disease costs of migraine disorders [8]. A recent European study found that there was an incremental burden due to migraine on HRQoL in terms of mental, physical, and health status. Both absenteeism and presenteeism, and the utilization of healthcare resources were also affected by migraine [6]. Similarly, US studies have found compromised HRQoL, and increased direct and indirect costs among migraine patients compared to non-migraine respondents [7]. However, few studies have investigated the burden of the illness in Japan [9, 10].

Japanese studies have shown that 32% of migraine patients reported severe to moderate impairment to social activities including cancellation of work and daily appointments [11]. Within a recall period of 3 months, 20.3% of migraine patients reported that they took time or days off from work and 27.9% reported being unable to do housework [12]. Additionally, migraine patients without aura had an average of 3.8 lost workdays and 2.8 days being unable to do housework [12]. During episodes of headaches, patients always (17.1%) or occasionally (43.4%) needed bed-rest [13]. On the other hand, although 74% of the patients reported severe headaches for which they desire for bed-rest, majority of them (68%) neither took days off from work nor skipped other social responsibilities [14].

Despite various reports summarized above, only limited studies used standard validated scales to measure the impact of migraine on Japanese migraine patients [10]. Additionally, there is lack of evidence demonstrating the burden to Japanese migraine patients compared to the general population without migraine.

The primary objective of this study was to estimate the humanistic and economic burden of migraine by comparison of HRQoL, work productivity and activity impairment (WPAI), and healthcare resource utilization (HRU) in individuals with migraine classified according to the International Classification of Headache Disorders 3rd edition (ICHD-3) [15] like criteria and having at least 4 monthly headache days (MHDs). The secondary objective was to examine the relationship between the humanistic and economic burden of migraine and sociodemographic factors, disease characteristics and comorbidities among Japanese migraine patients.

## Methods

This is a cross-sectional study using responses to the 2017 Japan National Health and Wellness Survey (NHWS). All respondents provided written informed consent. The survey was approved by the Pearl Pathways Institutional Review Board (IN, US). This analysis was granted exemption by the Public Health Research Foundation Ethical Review Committee.

### Study population

Potential respondents to the NHWS were identified through the web-based consumer panel Lightspeed Research (LSR). Panel members agreed to join the panel and received periodic invitations to online surveys. Panel members were recruited through co-registration with other internet panels, e-newsletter campaigns, and banner placements. Respondents for the 2017 NHWS were selected from the LSR panel members using a stratified random sampling framework with quotas based on age and gender from Japan national census data [16]. The NHWS is thereby a sample of the general population in Japan and representativeness has been validated and weighted against reliable sources including government agencies’ health statistics and unaffiliated third parties [17, 18].

### Exposure assessment

Migraine patients were defined by ICHD-3 like criteria, among respondents who self-reported experienced migraine in the past 12 months. ICHD-3 like criteria was created based on the diagnostic criteria from ICHD-3 [15] and the variables available in the NHWS data. ICHD-3 like criteria and the diagnostic criteria from ICHD-3 are summarized in Supplementary Table 1. Respondents having migraine without aura (including probable migraine without aura) or migraine with aura (including probable migraine with aura), according to ICHD-3 like criteria and having at least 4 MHDs (at least 4 headache days in the past 30 days) [19–21], were classified as migraine patients in this study. Respondents who reported no experience with migraine were classified as non-migraine respondents. Respondents experiencing pain caused by tension-type headache were not specifically excluded from the non-migraine respondents.

### Covariate assessment

Sociodemographic measures included age, gender, marital status, education, household income, health insurance, region and employment status. General health characteristics included smoking status, exercise behavior, alcohol use, body mass index (BMI) and Charlson Comorbidity Index (CCI) [22, 23]. Household income of 3,000,000 JPY (26,750 USD), 5,000,000 JPY (44,590 USD), and 8,000,000 JPY (71,350 USD) corresponded to around lowest 35th, 58th, and 80th percentile of annual household income in Japan [24]. CCI was derived by weighting the presence of twelve chronic conditions (self-reported diagnosis) and summed the result. A weight of one, two or six was given depending on the severity of the conditions. The minimum value of CCI is zero. The greater the total index score, the greater the comorbid burden on the respondent.

Migraine-specific questions were asked to all respondents who experienced migraine, which included: symptoms experienced due to migraine, number of years experiencing migraine, diagnosing physician, number of migraines in the past 30 days and in the past 6 months, number of headache days in the past 30 days, days of missed work due to migraine in the past 6 months, days of missed household activities due to migraine in the past 6 months, use of prescription medication to treat or prevent migraine. Migraine patients also answered the six-item Headache Impact Test-6 (HIT-6) [25]. HIT-6 is used to measure headache impact on the daily life of a patient, with higher score indicating larger impact on daily living [26].

### Outcome assessment

HRQoL was assessed by the short form 12 health survey version 2 (SF-12v2), which consists of 12 questions with three summary scores translated and validated for use in the Japanese population [27, 28]. The component summary scores, mental (MCS), physical (PCS) and role/social (RCS) were calculated using a norm-based scoring algorithm specific to the Japanese population [27, 29]. The SF-12v2 was also used to generate the short form 6 dimension (SF-6D) health state utilities by applying the SF-6D algorithm [30]. The SF-6D is a preference-based single index measure for health using general population values. Higher score indicates better quality of life.

The work productivity and activity impairment (WPAI) questionnaire was used to measure the impact of health on employment-related activities. It is a six-item validated instrument that consists of four metrics: absenteeism (percentage of work time missed because of one’s health in the past 7 days), presenteeism (percentage of impairment experienced because of one’s health while at work in the past 7 days), total work productivity impairment (an overall impairment estimate that is a combination of absenteeism and presenteeism), and total activity impairment (percentage of impairment in daily activities because of one’s health in the past 7 days) [31]. Only full-time, part-time, or self-employed respondents were included for absenteeism, presenteeism, and total work impairment assessment.

The HRU was quantified using the number of visits to healthcare providers (HCP) (including practitioner/family practitioners, internists, as well as specialists), number of emergency room (ER) visits, and number of hospitalizations in the past 6 months for the patient’s own medical condition. The HRU measured in this study was general and not limited to HRU specific to migraine.

Indirect costs due to absenteeism and presenteeism were calculated by integrating information from the WPAI questionnaire and hourly wage rates from the Japan Basic Survey on Wage Structure, 2017 [32], using the human capital method. For each employed respondent, hours lost due to absenteeism or presenteeism were multiplied by their estimated wage to estimate total weekly absenteeism costs, presenteeism costs, and indirect costs. Direct costs were estimated by multiplying unit costs for physician visits, emergency room visits, and hospitalizations obtained from Ministry of Health, Labor and Welfare, Japan [33] by the number of visits in the past 6 months. The unit costs were the average costs per person per healthcare facility visit for any conditions and treatments. Only direct costs related to the self-reported HRU were considered. Both indirect and direct costs were annualized.

### Statistical analysis

Demographic, general health characteristics, and migraine-specific characteristics were reported using counts and percentages for categorical variables and means and standard deviations (SDs) for continuous variables.

Comparisons between migraine patients and non-migraine respondents were performed using chi-square tests for categorical variables and one-way analysis of variance (ANOVA) for continuous variables, for demographic and general health characteristics, to understand the baseline differences between the two groups.

A matched control group of non-migraine respondents were created using propensity score matching (1:4 ratio of migraine patients to non-migraine respondents) using a greedy matching algorithm. Age, gender, marital status, education, household income, insurance, employment status, region, CCI, BMI, smoking status, alcohol use, and exercise were used in the matching. Matching was done for migraine patients with 4–7 MHDs, 8–14 MHDs, and more than 15 MHDs, respectively. Post-matching analyses were conducted to assess the balance of matching.

HRQoL, WPAI, HRU and costs were compared between migraine patients and matched non-migraine respondents. Multivariate analyses accounting for potential covariates were conducted using generalized linear models (GLMs). HRQoL scores (MCS, PCS, RCS, and SF-6D index) tended to be normally distributed. Normal distribution with identity link function was specified in the GLMs for these outcomes. WPAI, HRU and costs tended to have skewed distributions, and hence negative binomial distribution with log link function was specified. The covariates adjusted for in the GLMs included all sociodemographic and health characteristics described previously. Estimated adjusted means, 95% confidence intervals (CIs), and *p*-values were reported for each health outcome.

To identify factors associated with outcomes among migraine patients, multivariate analyses were conducted using sociodemographic factors, disease characteristics and comorbidities among migraine patients. Only variables found significant in the univariate analysis were included in the multivariate model.

Three sensitivity analyses were conducted. The main analysis was repeated for three groups including i) migraine group included definitive migraine patients only, and did not include probable migraine, ii) migraine patients who reported 1–3 MHDs as well as ≥4 MHDs, and iii) migraine patients with the additional “aura” symptom defined as the experience “See spots, flashing lights, or ‘heat waves’ before or during the migraine” or aura due to migraine (Supplementary Table 1).

For all analyses, *p* < 0.05 were considered statistically significant. All statistical analyses were performed using SPSS Version 22 [34] and R 3.5.1 [35].

## Results

### Participants

Out of 30,001 respondents to the 2017 Japan NHWS, a total of 4792 respondents self-reported experienced migraine in the past 12 months. 378 were classified as migraine patients according to the ICHD-3 like criteria and experienced at least 4 MHDs. 25,209 respondents reported not experienced migraine, of which 1512 respondents were matched non-migraine controls (Supplementary Figure 1).

### Demographic and clinical characteristic

After matching, no significant differences were observed between migraine patients and matched non-migraine respondents, except for CCI (Supplementary Table 2).

Among all migraine patients, more than half (55%) were diagnosed by primary care doctor/general practitioner/internist and 318 of them reported a migraine diagnosis received on average 14.6 years ago. About one third (36.2%) of migraine patients were currently taking prescription medication to treat or prevent migraine. In the past 30 days, migraine patients experienced 9.0 migraine episodes and 10.8 headache days on average. In the past 6 months, migraine patients on average had 46.0 migraine episodes, 2.8 days missed work and 6.7 days missed household activities due to migraine. Majority of the migraine patients (83.6%) were severely impacted based on the HIT-6 impact grade (Table 1).

**Table 1:** Self-reported migraine-related health characteristics among migraine patients

	Migraine patients with at least 4 monthly headache days	
**Continuous Variable**	**N**	**Mean (SD)**
**No. of years diagnosed of migraine**	318	14.6 (10.3)
**No. of migraines in the past 30 days**	378	9.0 (7.3)
**No. of migraines in the past 6 months**	378	46.0 (42.4)
**No. of headache days in the past 30 days**	378	10.8 (7.5)
**No. of days missed work due to migraines**	378	2.8 (15.4)
**No. of days missed household activities due to migraines**	378	6.7 (22.4)
**Categorical Variable**	**N**	**%**
**Monthly headache days**		
*≥ 15 days*	107	28.3%
*8–14 days*	88	23.3%
*4–7 days*	183	48.4%
**Diagnosing physician**		
*Primary care physician/GP/Internist*	175	55.0%
*Neurologist*	72	22.6%
*Other*	71	22.3%
**Currently taking prescription to treat or prevent migraine**	137	36.2%
**HIT-6 impact grades**		
*Little-to-no impact (HIT-6 score: 36–49)*	4	1.1%
*Moderate impact (HIT-6 score: 50–55)*	25	6.6%
*Substantial impact (HIT-6 score: 56–59)*	33	8.7%
*Severe impact (HIT-6 score: 60–78)*	316	83.6%
**Symptoms experienced**		
**Aura**	85	22.5%
**Moderate to severe pain**	240	63.5%
**Nausea and/or vomiting**	224	59.3%
**Pulsating, throbbing, or pounding pain**	329	87.0%
**Pain is worse on one side of your head or occurs on one side of your head only**	278	73.5%
**Pain is made worse by routine activities such as walking or climbing stairs**	196	51.9%
**Bothered by or unusually sensitive to light**	217	57.4%
**Bothered by or unusually sensitive to sound**	206	54.5%
**See spots, flashing lights, or “heat waves” before or during the migraine**	99	26.2%
**Lasts for at least four hours but not more than 72 h if untreated**	163	43.1%

Abbreviations: *SD* Standard deviation, *GP* General practitioner, *HIT-6* Headache Impact Test-6

The top three most common migraine symptoms were pulsating, throbbing, or pounding pain (87.0%), pain is worse on one side of head or occurs on one side of head only (73.5%) and moderate to severe pain (63.5%) (Table 1).

### Outcome assessment

#### HRQoL

Without adjustment, migraine patients had significantly lower PCS, MCS and RCS scores, compared to matched non-migraine respondents (Supplementary Table 3).

**Fig. 1:**
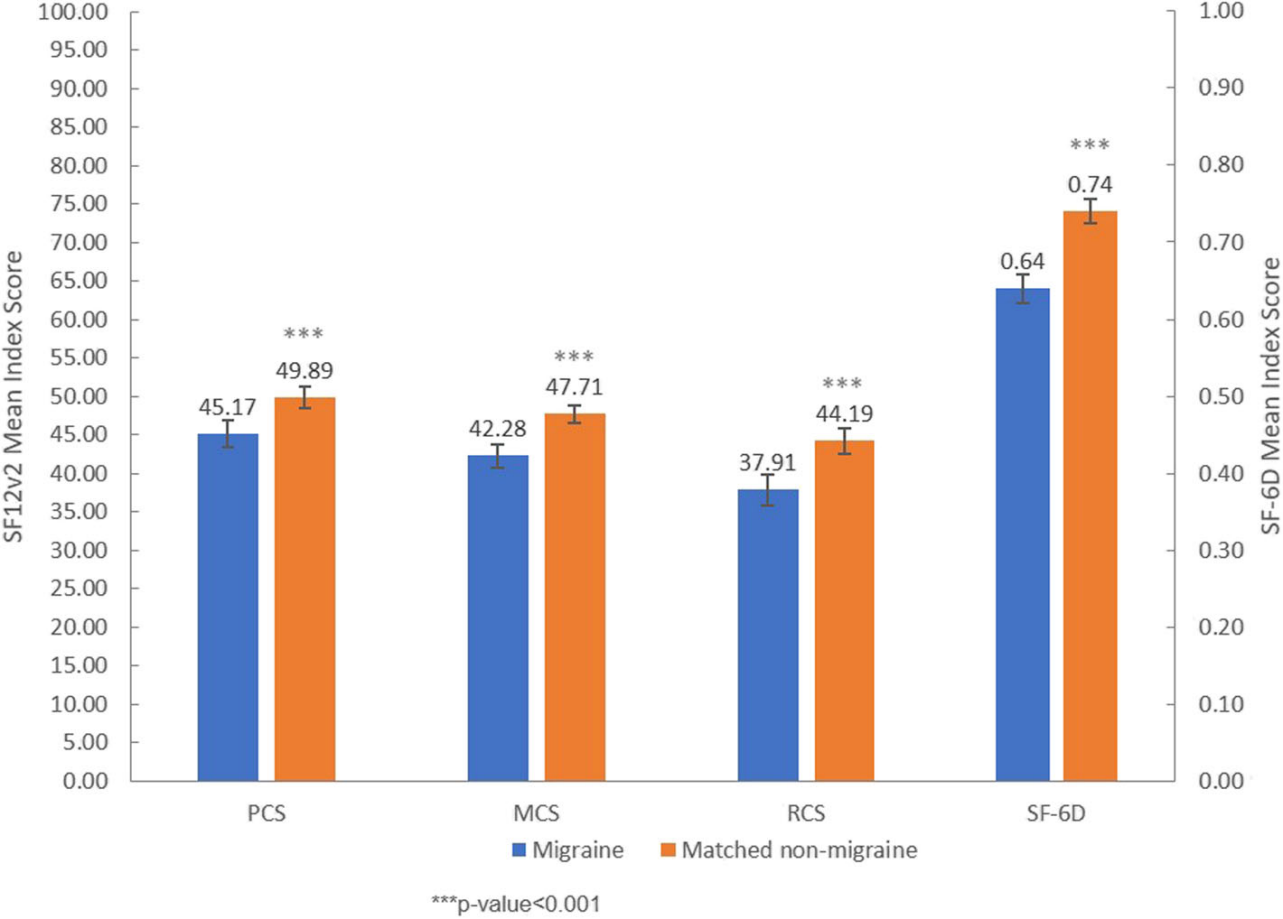
Adjusted means of PCS, MCS, RCS and SF-6D scores for migraine patients and matched non-migraine respondents of the Japan National Health and Wellness Survey 2017. Adjustment for following covariates: age, gender, CCI, marital status, education, household income, region, insurance type, employment status, BMI, smoking status, alcohol use and exercise behavior. Abbreviations: PCS = physical component summary score, MCS = mental component summary score, RCS = role/social component summary score, SF-6D = short form 6 dimension, SF-12v2 = short form 12 health survey version 2

After adjusting for age, gender, CCI, marital status, education, household income, region, insurance type, employment status, BMI, smoking status, alcohol use and exercise behavior, migraine patients had significantly lower PCS (45.17 vs. 49.89), MCS (42.28 vs. 47.71), RCS (37.91 vs. 44.19) and SF-6D index (0.64 vs. 0.74) than matched non-migraine respondents (all *p* < 0.001). The differences in adjusted means of PCS, MCS and RCS were all greater than 3 [36], which indicates a clinically significant decrease in HRQoL for migraine respondents (Fig. 1).

#### WPAI

Without adjustment, migraine patients experienced statistically significant higher impairment in absenteeism, presenteeism, total work productivity and total activity, compared to matched non-migraine respondents (Supplementary Table 3).

**Fig. 2:**
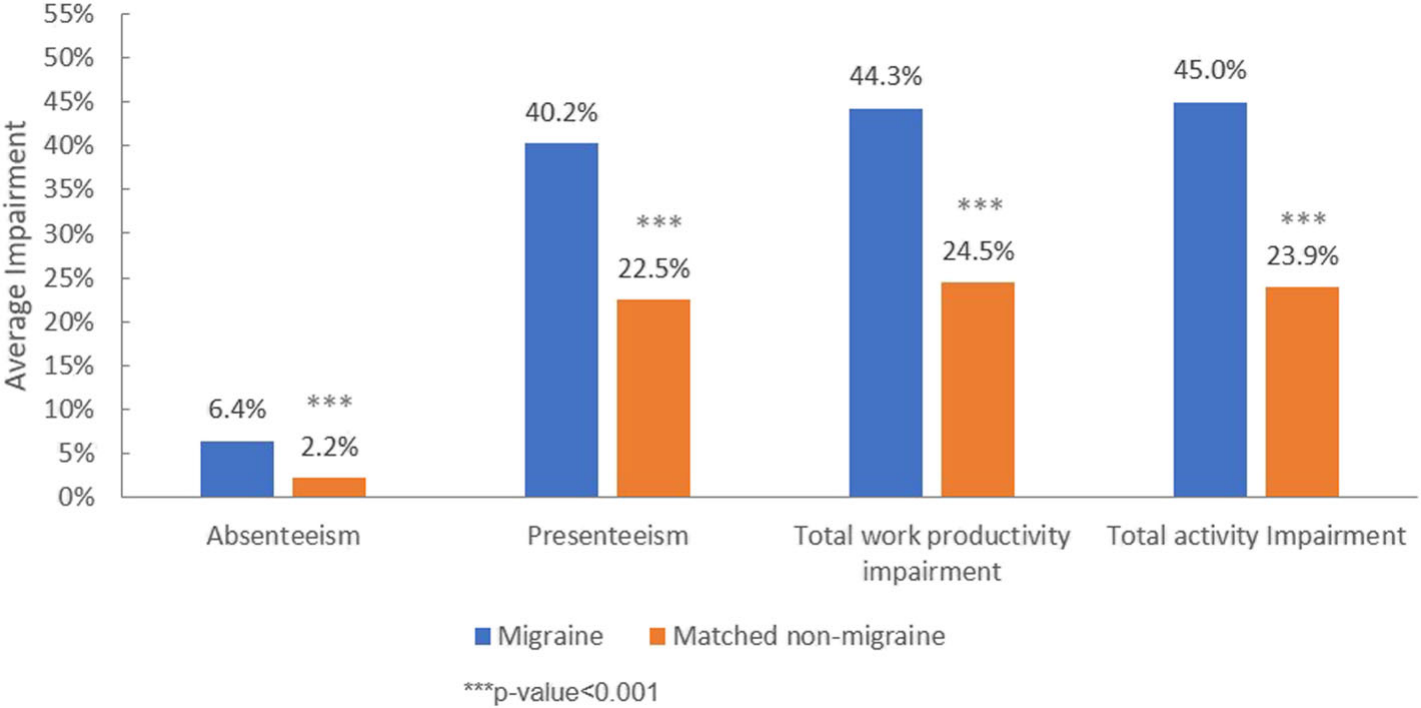
Adjusted means of work productivity and activity impairment (WPAI) for migraine patients and matched non-migraine respondents. Adjustment for following covariates: age, gender, CCI, marital status, education, household income, region, insurance type, employment status, BMI, smoking status, alcohol use and exercise behavior

After adjusting for the same covariates as mentioned above, migraine patients had significantly higher absenteeism (6.4% vs. 2.2%), presenteeism (40.2% vs. 22.5%), total work productivity impairment (44.3% vs. 24.5%) and total activity impairment (45.0% vs. 23.9%) than matched non-migraine respondents (all *p* < 0.001). The adjusted means of absenteeism, presenteeism, total work productivity impairment and total activity impairment of migraine patients were approximately twice as that of matched non-migraine respondents (Fig. 2).

#### HRU

**Fig. 3:**
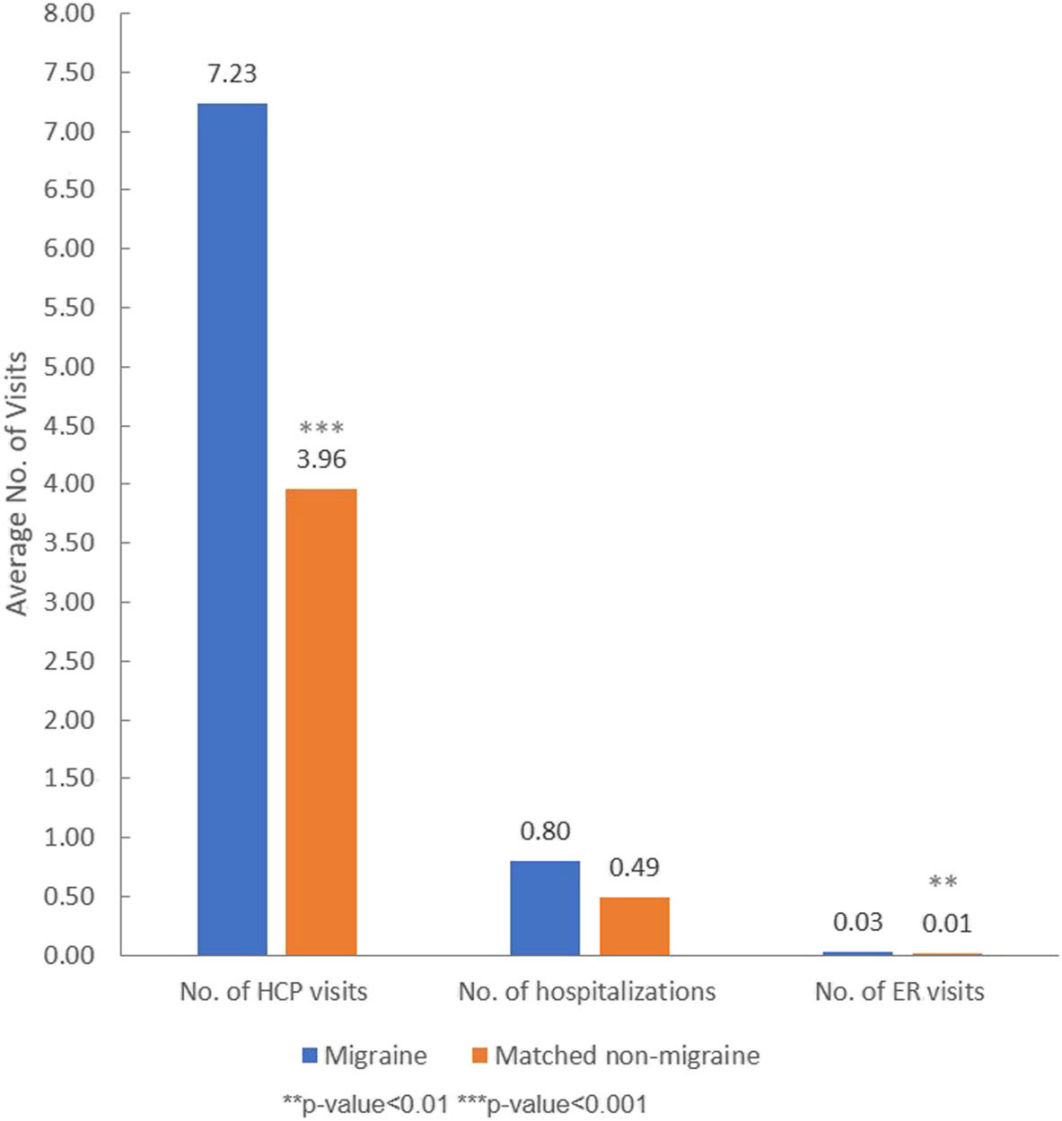
Adjusted means of the numbers of HCP visits, hospitalizations and ER visits for migraine patients and matched non-migraine respondents. Adjustment for following covariates: age, gender, CCI, marital status, education, household income, region, insurance type, employment status, BMI, smoking status, alcohol use and exercise behavior. Abbreviations: No. = number, HCP = healthcare providers, ER = emergency room

After adjustment for the same covariates as mentioned above, migraine patients had significantly more HCP visits (7.23 vs. 3.96) and ER visits (0.03 vs. 0.01) in the past 6 months, however the number of ER visits was very low in both groups. There was no significant difference between migraine patients and matched non-migraine respondents in terms of the number of hospitalizations in the past 6 months (Fig. 3).

#### Costs

**Fig. 4:**
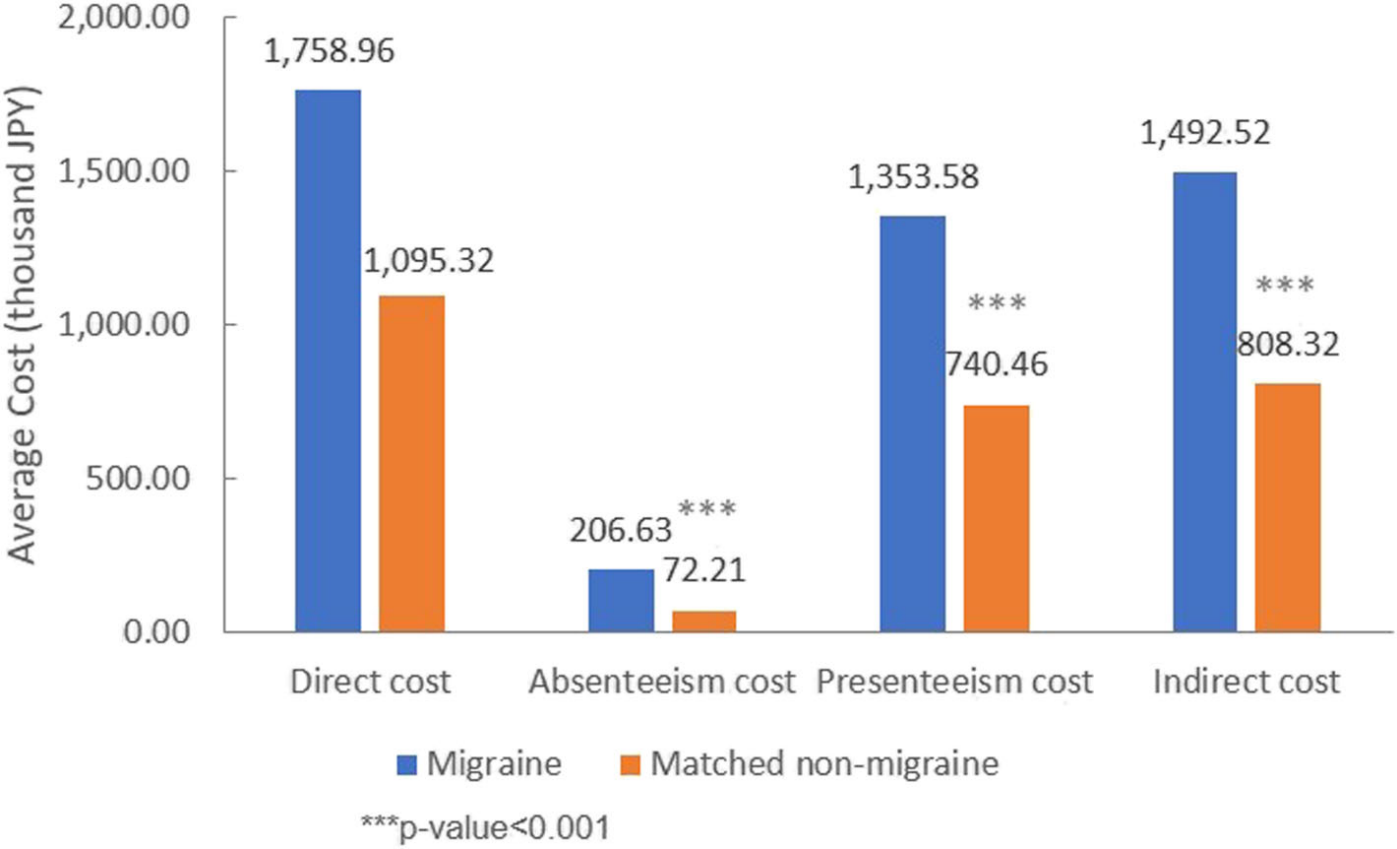
Adjusted means of annual direct cost and annual indirect cost for migraine patients and matched non-migraine respondents. Adjustment for following covariates: age, gender, CCI, marital status, education, household income, region, insurance type, employment status, BMI, smoking status, alcohol use and exercise behavior. Abbreviations: JPY = Japanese yen

After adjusting for the same covariates, it was estimated that migraine patients incurred significantly more annual indirect cost (1,492,520 JPY vs. 808,320 JPY) compared to matched non-migraine respondents. Both absenteeism cost (206,630 JPY vs. 72,210 JPY) and presenteeism cost (1,353,580 JPY vs. 740,460 JPY) were significantly higher among migraine patients than matched non-migraine respondents. However, the difference in the adjusted estimated annual direct cost between migraine patients and matched non-migraine respondents was not statistically significant (Fig. 4).

### Factors associated with outcomes

Among migraine-specific characteristics, having aura was negatively associated with PCS. MHDs were negatively associated with PCS, MCS, RCS and SF-6D score. Specifically, when MHDs were 15 days or more, compared to 4–7 MHDs, the negative associations with PCS, MCS, RCS, and SF-6D were all statistically significant (Table 2).

**Table 2:** Multivariate analyses of factors associated with humanistic and economic burden among Japanese migraine patients

**HRQoL Outcome Variable**	**Associated Variable**		**Coefficient [95% CI]**	**p**
**PCS** **(*N*** **= 378)**	CCI (per unit)		−2.83 [− 4.39, − 1.28]	< 0.001
	Aura	*Yes*	−3.97 [− 7.23, − 0.71]	0.017
		*No*	Reference	
	Monthly headache days	*≥15 days*	−6.99 [− 10.17, −3.80]	< 0.001
		*8–14 days*	−1.60 [− 4.97, 1.77]	
		*4–7 days*	Reference	
**MCS** **(*N*** **= 378)**	Marital status	*Married or living with partner*	Reference	0.020
		*Not Married / Decline to answer*	−2.33 [−4.30, −0.36]	
	Monthly headache days	*≥15 days*	−3.14 [−5.31, − 0.97]	0.004
		*8–14 days*	−3.08 [− 5.39, − 0.78]	
		*4–7 days*	Reference	
**RCS** **(*N*** **= 378)**	Age (per year)		0.22 [0.09, 0.34]	0.001
	Marital status	*Married or living with partner*	Reference	0.007
		*Not Married / Decline to answer*	−4.66 [−8.07, −1.25]	
	Currently employed	*Yes*	5.18 [2.02, 8.34]	0.001
		*No*	Reference	
	Monthly headache days	*≥15 days*	−3.85 [−7.42, −0.27]	0.048
		*8–14 days*	− 0.86 [−2.93, 4.65]	
		*4–7 days*	Reference	
**SF-6D** **(*N*** **= 378)**	Age (per year)		0.001 [0.001, 0.002]	0.007
	CCI (per unit)		−0.024 [−0.038, − 0.010]	0.001
	Marital status	*Married or living with partner*	Reference	0.007
		*Not Married / Decline to answer*	−0.037 [−0.064, − 0.010]	
	Currently employed	*Yes*	0.046 [0.021, 0.071]	< 0.001
		*No*	Reference	
	Monthly headache days	*≥15 days*	−0.064 [−0.092, − 0.036]	< 0.001
		*8–14 days*	−0.021 [− 0.051, 0.009]	
		*4–7 days*	Reference	
**WPAI Outcome Variable**	**Associated Variable**		**OR [95% CI]**	***p*-value**
**Absenteeism** **(*N*** **= 208)**	Pain is worse on one side of your head or occurs on one side of your head only	*Yes*	2.15 [1.17, 3.94]	0.014
		*No*	Reference	
**Total activity impairment** **(*N*** **= 207)**	CCI (per unit)		1.13 [1.03, 1.25]	0.013
	Marital status	*Married or living with partner*	Reference	0.023
		*Not Married / Decline to answer*	1.20 [1.03, 1.41]	
	Level of education	*Completed university education*	Reference	0.002
		*No university education*	0.82 [0.69, 0.96]	
		*Decline to answer*	0.67 [0.53, 0.86]	
	Region	*Hokkaido*	Reference	0.032
		*Tohoku*	0.63 [0.39, 1.03]	
		*Kanto*	0.84 [0.55, 1.28]	
		*Chubu*	0.75 [0.48, 1.17]	
		*Kinki*	0.84 [0.54, 1.32]	
		*Chugoku*	0.66 [0.38, 1.14]	
		*Shikoku*	0.46 [0.26, 0.82]	
		*Kyushu/Okinawa*	0.66 [0.41, 1.05]	
	Currently employed	*Yes*	0.76 [0.65, 0.88]	< 0.001
		*No*	Reference	
	Monthly headache days	*≥15 days*	1.45 [1.22, 1.73]	< 0.001
		*8–14 days*	1.11 [0.92, 1.35]	
		*4–7 days*	Reference	
**HRU Outcome Variable**	**Associated Variable**		**OR [95% CI]**	***p*-value**
**No. of HCP visits in the past 6 months** **(*N*** **= 378)**	Age (per year)		1.02 [1.01, 1.03]	0.002
	CCI (per unit)		1.45 [1.19, 1.76]	< 0.001
	Monthly headache days	*≥15 days*	2.10 [1.58, 2.79]	< 0.001
		*8–14 days*	1.42 [1.05, 1.92]	
		*4–7 days*	Reference	
**No. of hospitalizations in the past 6 months** **(*N*** **= 378)**	CCI (per unit)		4.29 [1.61, 11.48]	0.004
	Level of education	*Completed university education*	Reference	0.006
		*Not*	0.12 [0.03, 0.45]	
		*Decline to answer*	0.44 [0.06, 3.20]	
	See spots, flashing lights, or “heat waves” before or during the migraine	*Yes*	4.59 [1.08, 19.54]	0.039
		*No*	Reference	
	Monthly headache days	*≥15 days*	7.88 [1.90, 32.76]	0.001
		*8–14 days*	12.03 [3.02, 47.89]	
		*4–7 days*	Reference	
**Cost Outcome Variable**	**Associated Variable**		**OR [95% CI]**	**p**
**Absenteeism Cost** **(*N*** **= 208)**	Gender	*Male*	Reference	0.022
		*Female*	0.47 [0.25, 0.90]	
	Pain is worse on one side of your head or occurs on one side of your head only	*Yes*	2.19 [1.17, 4.11]	0.014
		*No*	Reference	
**Presenteeism Cost** **(*N*** **= 219)**	Gender	*Male*	Reference	< 0.001
		*Female*	0.56 [0.43, 0.74]	
**Indirect Cost** **(*N*** **= 207)**	Gender	*Male*	Reference	< 0.001
		*Female*	0.56 [0.43, 0.72]	

Abbreviations: *HRQoL* Health-related quality of life, *WPAI* Work productivity and activity impairment, *HRU* Healthcare resource utilization, *PCS* Physical component summary, *MCS* Mental component summary, *RCS* Role/social component summary, *HCP* Healthcare providers, *ER* Emergency room, *CCI* Charlson comorbidity index, *RR* Relative risk, *OR* Odds ratio, *CI* Confidence interval

Having 15 or more MHDs, compared to 4–7 MHDs, was associated with 1.45 [95% CI: 1.22, 1.73] times increased total activity impairment. Additionally, having worse pain in one side of the head was associated with 2.15 [1.17, 3.94] times increased absenteeism (Table 2).

More MHDs were also significantly associated with higher number of HCP visits and hospitalizations. Compared to those with 4–7 MHDs, migraine patients with 8–14 MHDs and 15 or more MHDs had more hospitalizations in the past 6 months (Table 2).

More details of the other sociodemographic variables that were associated with HRQoL, WPAI and HRU among migraine patients can be found in Table 2.

### Sensitivity analysis

Results from all three sensitivity analyses were consistent with the main results; i) when the migraine group included definitive migraine patients only, and did not include probable migraine, ii) when migraine patients included those who reported 1–3 days of headache in the past 30 days (MHDs) as well as ≥4 MHDs, and iii) when migraine patients with the additional “aura” symptom as defined in ICHD-3 like criteria, that is, experience “See spots, flashing lights, or ‘heat waves’ before or during the migraine” or aura due to migraine (Supplementary Table 1) were included. Details are available upon request.

## Discussion

This study was a large-scale broad examination of the humanistic and economic burden among Japanese migraine patients defined by ICHD-3 like criteria (Supplementary Table 1), that provides up-to-date results based on the 2017 NHWS data. We found that Japanese patients with migraine (with or without aura and with at least 4 MHDs) experienced significantly higher humanistic and economic burden in terms of decreased HRQoL, and increased WPAI, HRU and indirect costs, compared to matched non-migraine respondents. The differences in HRQoL components were statistically and clinically significant (difference > 3 points) [36], and the decrease in WPAI and increase in HRU among migraine patients were about two times that of matched non-migraine respondents. The increased indirect cost was primarily driven by increased presenteeism costs, however the difference in direct cost was not statistically significant.

One strength of this study was the use of a validated instrument WPAI to quantify the work impairment among migraine patients in comparison to the non-migraine population in Japan, as few other studies have used validated tools for work impairment assessment [9–14]. Also, previous studies have primarily focused on analyzing HRU and costs related to migraine within the migraine population [37, 38]; hence, our study provided a novel point of view in the burden of the healthcare system utilization and costs of migraine in comparison with the non-migraine population.

Unexpectedly, no statistically significant difference in direct costs between migraine patients and non-migraine respondents was observed in this study. This is supported by the findings that more than 60% of migraine patients did not consult a physician [12]. It should be noted that the direct costs in this study were derived primarily from the number of physician visits, emergency room visits, and hospitalizations, which were self-reported from the patients. Over-the-counter medication costs and prescription medication costs for migraine were also not included in the calculation which could lead to potential under-estimation of the direct costs. Additionally, indirect costs were significantly lower among non-migraine respondents, indicating that the poorer QoL and work productivity among migraine patients have a costly impact on the Japanese society. These results indicate that there is an unmet need for future medical treatments to improve QoL and work productivity, and thereby lower the burden and indirect costs associated with migraine.

A retrospective, observational cohort study in the US identified that migraine patients incurred significantly higher indirect cost due to higher productivity loss, as well as direct cost due to higher level of healthcare utilization, compared to matched patients without migraine [39]. Migraine patients in the US study were identified when they received their migraine diagnoses and/or medications, while we also captured patients who experienced symptoms of migraine but yet consulted a physician. Although the impact on direct cost seemed not as substantial in Japan among this group of patients, impairment in the QoL, work productivity, and indirect costs were significant to these patients and to the overall Japanese society. It is thus crucial to encourage these patients to seek formal medical attention and reduce their burden of disease through proper medical treatment.

A similar study on migraine patients in Europe showed a decrease in HRQoL and higher work impairment among migraine patients compared to non-migraine respondents [6]. The lower reported MCS (5.43 vs. 7.13) and absenteeism (6.4% vs. 14.4%), but higher total work productivity impairment (45.0% vs. 38.7%) among Japanese migraine patients compared to patients in Europe could be due to the “Gaman” cultural concept in Japan where individuals were encouraged to show patience, perseverance and tolerance when facing unexpected or difficult situations [40].

The underlying differences, especially the difference in the prevalence of migraine across the regions should be noted when comparing across regions. The prevalence was reported to be around 6% to 8% in Japan [9, 11], 15% in Europe [41, 42] and in the US [43]. A recent literature review revealed that there was a low level of disease awareness and use of prescription medication for migraine in East Asia [9]. Among Japanese migraine patients, approximately two thirds received acute treatment only, 15% received prophylactic treatment and 22% received no treatment [44]. This further adds to the importance of raising awareness for patients with migraine to seek physician advice and appropriate medical interventions.

This current study reported that an increased number of MHDs was associated with worse health outcomes (physical, mental and role/social health) and increased HRU (Table 2), especially among patients with chronic migraine (≥15 MHDs). This highlights the need for more effective treatments, especially for the group of patients suffering from chronic migraine [9, 45], to reduce the MHDs and thus improve health outcomes for migraine patients. Health outcomes of chronic migraine patients and episodic migraine patients were not directly compared in this study which can be further investigated in future studies.

Another US study discovered that depression was highly correlated with productivity loss in the headache population, and thus concluded that early recognition and treatment of comorbid depression could improve QoL and productivity as well as decrease healthcare resource use [46]. It highlighted the importance to understand the common comorbidities of migraine in Japan that is currently lacking, and how controlling these comorbidities might help to improve the overall health outcomes of patients with migraine.

Limitations of this study include that the NHWS data is cross-sectional and no causal relationships between migraine status and outcomes can be assumed. Respondents self-reported all data and verification of these could not be performed and recall bias could not be excluded. Due to availability of self-reported information, the ICHD-3 like criteria (Supplementary Table 1) were created. Although reflecting ICHD-3 criteria [15] closely, discrepancies could not be avoided. Tension-type headache was not specifically excluded from this study and might have led to variations in the number of monthly headache days. It is also of interest to further investigate patients with tension-type headache to identify the burden and unmet needs of this group. Treatment details for migraine will be able to provide more insights regarding the burden but were not captured in the current study. Future studies are warranted to further understand the treatment landscape and treatment costs of migraine patients in Japan. As the study focused on comparison of patients with migraine and with respondents without migraine, WPAI, HRU and costs measured in this study were not migraine-specific and should be interpreted only relative to the matched non-migraine respondents. In addition, although NHWS is broadly representative of the Japanese adult population, the extent to which migraine patients are representative of the larger population is unknown.

## Conclusions

In conclusion, migraine patients in Japan experience an incremental burden compared to matched non-migraine respondents in terms of decreased HRQoL, almost twice as impaired productivities, and almost two-fold higher self-reported overall HCP visits, and higher overall indirect costs. Increased number of MHDs among Japanese migraine patients were associated with worse HRQoL and increased HRU. This demonstrates that better care is needed for Japanese migraine patients.

## Supplementary information

**Additional file 1: Supplementary Table 1.** ICHD-3 like criteria used in this study and ICHD-3 diagnostic criteria [14].

**Additional file 2: Supplementary Table 2.** Demographic and clinical characteristics between migraine patients and matched non-migraine respondents.

**Additional file 3: Supplementary Table 3.** Comparison of HRQoL, WPAI, HRU and costs between migraine patients and matched non-migraine respondents.

**Additional file 4: Supplementary Figure 1.** Flow chart of study sample selection.

## Data Availability

The data that support the findings of this study are available from Kantar, but restrictions apply to the availability of these data, which were used under license for the current study, and so are not publicly available. Data are however available from the authors upon reasonable request and with permission of Kantar.

## Abbreviations

ANOVA: Analysis of variance
BMI: Body mass index
CCI: Charlson Comorbidity Index
CI: Confidence interval
ER: Emergency room
GLM: Generalized linear model
HCP: Healthcare provider
HIT-6: Headache Impact Test-6
HRQoL: Health-related quality of life
HRU: Healthcare resource utilization
ICHD-3: International Classification of Headache Disorders 3rd edition
JPY: Japanese yen
LSR: Lightspeed Research
MCS: Mental component summary
MHDs: Monthly headache days
NHWS: National Health and Wellness Survey
PCS: Physical component summary
RCS: Role/social component summary
SF-12v2: Short form 12 health survey version 2
SF-6D: Short form 6 dimension
SD: Standard deviation
WPAI: Work productivity and activity impairment
YLD: Years lived with disability
