# Supplementary material for: Burden of migraine among Japanese patients: a cross-sectional National Health and Wellness Survey
Source: J Headache Pain. 2020 Sep 10;21(1):110. doi: 10.1186/s10194-020-01180-9 (PMC7488335; doi:10.1186/s10194-020-01180-9)
Supplement: Supplementary file 1 — Additional file 1: Supplementary Table 1. ICHD-3 like criteria used in this study and ICHD-3 diagnostic criteria [14]. [file 10194_2020_1180_MOESM1_ESM.docx]

**Supplementary Table 1. ICHD-3 like criteria used in this study and ICHD-3 diagnostic criteria** [14]

|  | **ICHD-3 like criteria used in this study** | **ICHD-3 diagnostic criteria** |
| --- | --- | --- |
| **Migraine without aura** | Fulfilling all the below criteria A-D among patients who self-reported of experience migraine in the past 12 months:  A. At least five migraines in the past 6 months OR self-reported physician diagnosis of migraine  B. Migraine lasts for at least four hours but not more than 72 hours if untreated  C. Experience at least two of the following due to migraine:  1. Pain is worse on one side of your head or occurs on one side of your head only  2. Pulsating, throbbing, or pounding pain  3. Moderate to severe pain  4. Pain is made worse by routine  D. Experience at least one of the following due to migraine:  1. Nausea and/or vomiting  2. Bothered by or unusually sensitive to light  3. Bothered by or unusually sensitive to sound | A. At least five attacks fulfilling criteria B-D  B. Headache attacks lasting 4-72 hr (untreated or unsuccessfully treated)  C. Headache has at least two of the following four characteristics:   1. unilateral location 2. pulsating quality 3. moderate or severe pain intensity 4. aggravation by or causing avoidance of routine physical activity (eg, walking or climbing stairs)   D. During headache at least one of the following:   1. nausea and/or vomiting 2. photophobia and phonophobia   E. Not better accounted for by another ICHD-3 diagnosis |
| **Probable Migraine without aura** | All but one of above criteria A–D among those with migraine in the past 12 months | A. Attacks fulfilling all but one of criteria A–D for 1.1 Migraine without aura  B. Not fulfilling ICHD-3 criteria for any other headache disorder  C. Not better accounted for by another ICHD-3 diagnosis. |
| **Migraine with aura** | Fulfilling both of below criteria A–B among patients who self-reported of experience migraine in the past 12 months:  A. At least two migraines in the past 6 months OR self-reported physician diagnosis of migraine  B. Experience “See spots, flashing lights, or ‘heat waves’ before or during the migraine” due to migraine | A. At least two attacks fulfilling criteria B and C  B. One or more of the following fully reversible aura symptoms:   1. visual 2. sensory 3. speech and/or language 4. motor 5. brainstem 6. retinal   C. At least three of the following six characteristics:   1. at least one aura symptom spreads gradually over ≥5 minutes 2. two or more aura symptoms occur in succession 3. each individual aura symptom lasts 5-60 minutes 4. at least one aura symptom is unilateral 5. at least one aura symptom is positive 6. the aura is accompanied, or followed within 60 minutes, by headache   D. Not better accounted for by another ICHD-3 diagnosis. |
| **Probable Migraine with aura** | Fulfilling both of below criteria A–B among patients who self-reported of experience migraine in the past 12 months:  A. No more than one migraine in the past 6 months WITHOUT self-reported physician diagnosis of migraine  B. Experience “See spots, flashing lights, or ‘heat waves’ before or during the migraine” due to migraine | A. Attacks fulfilling all but one of criteria A–C for 1.2 Migraine with aura or any of its subtypes  B. Not fulfilling ICHD-3 criteria for any other headache disorder  C. Not better accounted for by another ICHD-3 diagnosis. |
