# Supplementary material for: Burden of migraine among Japanese patients: a cross-sectional National Health and Wellness Survey
Source: J Headache Pain. 2020 Sep 10;21(1):110. doi: 10.1186/s10194-020-01180-9 (PMC7488335; doi:10.1186/s10194-020-01180-9)
Supplement: Supplementary file 2 — Additional file 2: Supplementary Table 2. Demographic and clinical characteristics between migraine patients and matched non-migraine respondents. [file 10194_2020_1180_MOESM2_ESM.docx]

**Supplementary Table 2. Demographic and clinical characteristics between migraine patients and matched non-migraine respondents**

|  | | **Migraine with at least 4 monthly headache days** | | **Matched non-migraine** | | **p** | **Standardized mean difference** |
| --- | --- | --- | --- | --- | --- | --- | --- |
| **Continuous Variable** | | **N** | **Mean (SD)** | **N** | **Mean (SD)** |  |  |
| **Age** | | 378 | 41.6 (12.7) | 1,512 | 41.2 (15.0) | 0.65 | 0.028 |
| **Charlson Comorbidity Index** | | 378 | 0.24 (0.86) | 1,512 | 0.13 (0.57) | 0.004 | 0.146 |
| **Categorical Variable** | | **N** | **%** | **N** | **%** |  |  |
| **Gender** | *Male* | 76 | 20.1% | 321 | 21.2% | 0.63 | 0.028 |
|  | *Female* | 302 | 79.9% | 1,191 | 78.8% |  |  |
| **Marital status** | *Married or living with partner* | 185 | 48.9% | 750 | 49.6% | 0.82 | 0.013 |
|  | *Not married / Decline to answer* | 193 | 51.1% | 762 | 50.4% |  |  |
| **Level of education** | *Completed university education* | 156 | 41.3% | 644 | 42.6% | 0.84 | 0.033 |
|  | *No completed university education* | 176 | 46.6% | 679 | 44.9% |  |  |
|  | *Decline to answer* | 46 | 12.2% | 189 | 12.5% |  |  |
| **Household income** | *<3,000,000 JPY* | 74 | 19.6% | 293 | 19.4% | 1.00 | 0.025 |
|  | *3,000,000 to <5,000,000 JPY* | 102 | 27.0% | 415 | 27.4% |  |  |
|  | *5,000,000 to <8,000,000 JPY* | 93 | 24.6% | 362 | 23.9% |  |  |
|  | *8,000,000 JPY or more* | 56 | 14.8% | 220 | 14.6% |  |  |
|  | *Decline to answer* | 53 | 14.0% | 222 | 14.7% |  |  |
| **Region** | *Hokkaido* | 12 | 3.2% | 50 | 3.3% | 1.00 | 0.035 |
|  | *Tohoku* | 25 | 6.6% | 99 | 6.5% |  |  |
|  | *Kanto* | 149 | 39.4% | 587 | 38.8% |  |  |
|  | *Chubu* | 55 | 14.6% | 226 | 14.9% |  |  |
|  | *Kinki* | 71 | 18.8% | 287 | 19.0% |  |  |
|  | *Chugoku* | 16 | 4.2% | 56 | 3.7% |  |  |
|  | *Shikoku* | 12 | 3.2% | 48 | 3.2% |  |  |
|  | *Kyushu/Okinawa* | 38 | 10.1% | 159 | 10.5% |  |  |
| **Insurance type** | *National health insurance* | 197 | 52.1% | 789 | 52.2% | 0.89 | 0.028 |
|  | *Social insurance* | 169 | 44.7% | 682 | 45.1% |  |  |
|  | *Other* | 12 | 3.2% | 41 | 2.7% |  |  |
| **Currently employed** | *Yes* | 227 | 60.1% | 906 | 59.9% | 0.96 | 0.003 |
|  | *No* | 151 | 39.9% | 606 | 40.1% |  |  |
| **BMI category** | *Underweight (BMI<18.5)* | 69 | 18.3% | 281 | 18.6% | 0.92 | 0.040 |
|  | *Normal weight (18.5≤BMI<25) / Decline to answer* | 236 | 62.4% | 918 | 60.7% |  |  |
|  | *Pre-obese (25≤BMI<30)* | 56 | 14.8% | 243 | 16.1% |  |  |
|  | *Obese (BMI*≥*30)* | 17 | 4.5% | 70 | 4.6% |  |  |
| **Smoking status** | *Never* | 211 | 55.8% | 827 | 54.7% | 0.90 | 0.028 |
|  | *Former* | 79 | 20.9% | 316 | 20.9% |  |  |
|  | *Current* | 88 | 23.3% | 369 | 24.4% |  |  |
| **Alcohol use** | *Abstain* | 153 | 40.5% | 632 | 41.8% | 0.64 | 0.027 |
|  | *Currently consume alcohol* | 225 | 59.5% | 880 | 58.2% |  |  |
| **Vigorous exercise in past 30 days** | *Yes* | 158 | 41.8% | 627 | 41.5% | 0.91 | 0.007 |
|  | *No* | 220 | 58.2% | 885 | 58.5% |  |  |

Abbreviations: JPY = Japanese yen, SD = standard deviation, BMI = body mass index.
