# Supplementary material for: Burden of migraine among Japanese patients: a cross-sectional National Health and Wellness Survey
Source: J Headache Pain. 2020 Sep 10;21(1):110. doi: 10.1186/s10194-020-01180-9 (PMC7488335; doi:10.1186/s10194-020-01180-9)
Supplement: Supplementary file 3 — Additional file 3: Supplementary Table 3. Comparison of HRQoL, WPAI, HRU and costs between migraine patients and matched non-migraine respondents. [file 10194_2020_1180_MOESM3_ESM.docx]

**Supplementary Table 3. Comparison of HRQoL, WPAI, HRU and costs between migraine patients and matched non-migraine respondents**

|  | | **Migraine with at least 4 monthly headache days** | | **Matched non-migraine** | | **p** |
| --- | --- | --- | --- | --- | --- | --- |
| **Outcome Variable** | | **N** | **Mean (SD)** | **N** | **Mean (SD)** |  |
| **HRQoL** | SF-12v2: PCS | 378 | 46.85 (14.09) | 1,512 | 51.90 (11.04) | < 0.001 |
|  | SF-12v2: MCS | 378 | 42.26 (9.28) | 1,512 | 47.71 (10.12) | < 0.001 |
|  | SF-12v2: RCS | 378 | 39.79 (15.83) | 1,512 | 46.20 (13.22) | < 0.001 |
|  | SF-6D health utilities | 378 | 0.65 (0.13) | 1,512 | 0.75 (0.13) | < 0.001 |
| **WPAI** | Absenteeism | 208 | 7.7% (16.1%) | 844 | 3.9% (14.5%) | < 0.001 |
|  | Presenteeism | 219 | 37.1% (29.0%) | 869 | 20.7% (25.3%) | < 0.001 |
|  | Total work productivity impairment | 207 | 39.9% (29.6%) | 838 | 22.3% (26.9%) | < 0.001 |
|  | Total activity Impairment | 378 | 42.3% (29.6%) | 1,512 | 22.4% (25.9%) | < 0.001 |
| **HRU** | No. of HCP visits in the past 6 months | 378 | 8.44 (13.73) | 1,512 | 4.32 (7.24) | < 0.001 |
|  | No. of hospitalization in the past 6 months | 378 | 0.90 (8.29) | 1,512 | 0.38 (2.30) | 0.033 |
|  | No. of ER visits in the past 6 months | 378 | 0.10 (0.50) | 1,512 | 0.05 (0.38) | 0.020 |
| **Annual Costs** | Direct Cost (thousand JPY) | 378 | 2,089.24 (1,8017.39) | 1,512 | 896.43 (4977.44) | 0.024 |
|  | Indirect cost (thousand JPY) | 207 | 1,352.11 (1,080.96) | 838 | 703.62 (865.56) | < 0.001 |
|  | Absenteeism cost (thousand JPY) | 208 | 254.87 (509.15) | 844 | 118.21 (447.23) | < 0.001 |
|  | Presenteeism cost (thousand JPY) | 219 | 1,251.96 (1,023.44) | 869 | 660.46 (826.59) | < 0.001 |

Abbreviations: HRQoL = health-related quality of life, WPAI = work productivity and activity impairment, HRU = healthcare resource utilization, PCS = physical component summary score, MCS = mental component summary score, RCS = role/social component summary score, SF-12v2 = short form 12 health survey version 2, SF-6D = short form 6 dimension, HCP = healthcare providers, ER = emergency room, JPY = Japanese yen, SD = standard deviation.
