# Supplementary material for: Burden of migraine among Japanese patients: a cross-sectional National Health and Wellness Survey
Source: J Headache Pain. 2020 Sep 10;21(1):110. doi: 10.1186/s10194-020-01180-9 (PMC7488335; doi:10.1186/s10194-020-01180-9)
Supplement: Supplementary file 4 — Additional file 4: Supplementary Figure 1. Flow chart of study sample selection. [file 10194_2020_1180_MOESM4_ESM.docx]

**Supplementary Figure 1. Flow chart of study sample selection**


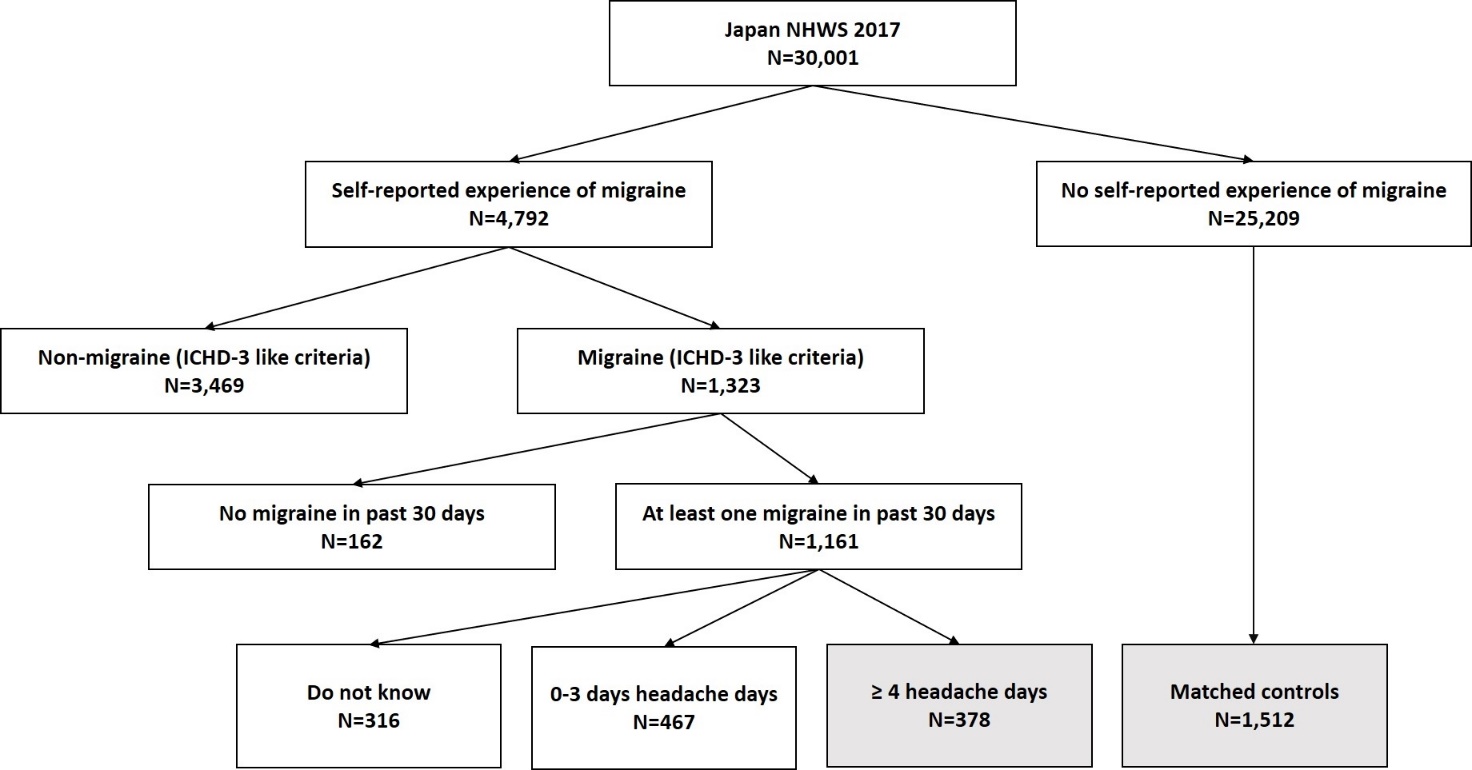


Abbreviations: NHWS=National Health and Wellness Survey, ICHD-3=International Criteria of Headache Disorders, 3^rd^ edition.
